# Extracorporeal life support in cardiogenic shock: indications and management in current practice

**DOI:** 10.1007/s12471-018-1073-9

**Published:** 2018-01-18

**Authors:** C. L. Meuwese, F. Z. Ramjankhan, S. A. Braithwaite, N. de Jonge, M. de Jong, M. P. Buijsrogge, J. G. D. Janssen, C. Klöpping, J. H. Kirkels, D. W. Donker

**Affiliations:** 1 Department of Cardiology, University Medical Center Utrecht, Utrecht, The Netherlands; 2 Department of Cardiothoracic Surgery, University Medical Center Utrecht, Utrecht, The Netherlands; 3 Department of Anesthesiology, University Medical Center Utrecht, Utrecht, The Netherlands; 4 Department of Intensive Care Medicine, University Medical Center Utrecht, Utrecht, The Netherlands

**Keywords:** Cardiogenic shock, Short-term cardiac mechanical support, Extracorporeal membrane oxygenation (ECMO), Extracorporeal life support (ECLS)

## Abstract

Veno-arterial extracorporeal life support (VA-ECLS) provides circulatory and respiratory stabilisation in patients with severe refractory cardiogenic shock. Although randomised controlled trials are lacking, the use of VA-ECLS is increasing and observational studies repeatedly have shown treatment benefits in well-selected patients. Current clinical challenges in VA-ECLS relate to optimal management of the individual patient on extracorporeal support given its inherent complexity. In this review article we will discuss indications, daily clinical management and complications of VA-ECLS in cardiogenic shock refractory to conventional treatment strategies.

## Introduction

Although survival seems to have improved during recent years, cardiogenic shock continues to carry a poor prognosis. After myocardial infarction, mortality rates range from 50 to 75% despite urgent coronary revascularisation [1]. In non-ischaemic cardiac disease, cardiogenic shock carries a comparably unfavourable outcome [2].

Cardiogenic shock is encountered in 5–10% of patients with ST-elevation myocardial infarction (STEMI) and up to 3% of those with non-STEMI [2, 3]. In non-ischaemic cardiomyopathy, shock may develop as an acute or acute on chronic manifestation. In the Netherlands, around 28,000 individuals suffer from acute myocardial infarction annually and the number of chronic heart failure patients currently approximates 230,000 and is increasing [4]. This implicates that, potentially, over 2,000 individuals present with cardiogenic shock annually.

The shown absence of a survival benefit of the intra-aortic balloon pump (IABP) compared to conventional treatment in post-infarction cardiogenic shock without mechanical complications in the SHOCK II trial [5] in conjunction with previous evidence [6], has led to a downgrading of guideline recommendations for IABP use in Europe from Class I [level of evidence: C] to Class III [A] [7]. This critical evaluation of the IABP has created a new impetus to reflect on the role of alternative short-term mechanical support devices [6], such as veno-arterial extracorporeal life support (VA-ECLS), which can provide partial or full circulatory support [8]. In addition, VA-ECLS can provide respiratory support in patients suffering from severe, combined cardiac and pulmonary failure. Although randomised trials evaluating VA-ECLS in cardiogenic shock are still lacking [9], observational studies have indicated beneficial effects in patients with cardiogenic shock in acute on chronic heart failure and cardiac arrest [10].

In this review we will discuss current indications, clinical management and potential complications of VA-ECLS in patients with cardiogenic shock refractory to conventional treatment.

## Veno-arterial extracorporeal life support, basic concepts

**Fig. 1:**
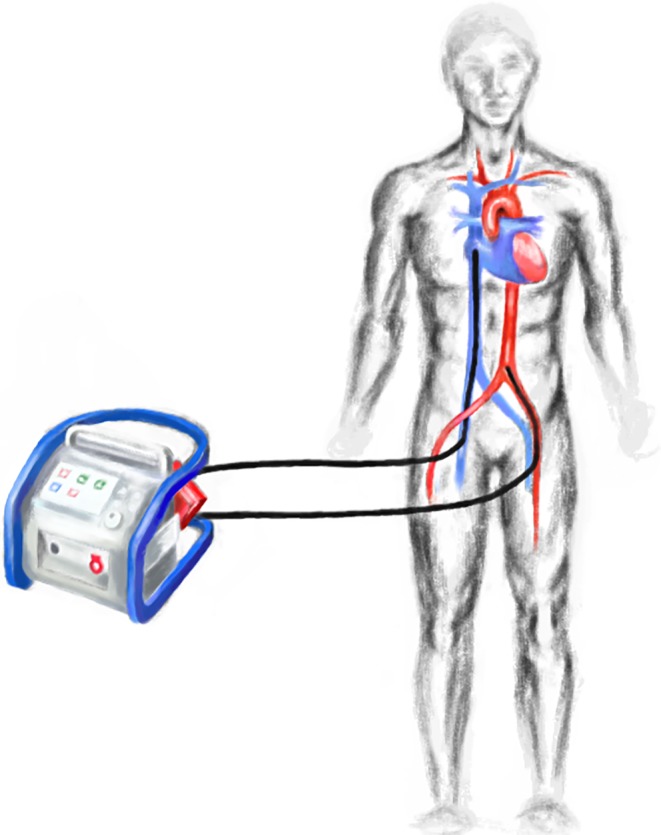
Schematic illustration of a VA-ECLS system

In a VA-ECLS circuit, central venous blood is drained, relayed through an extracorporeal pump and oxygenator and then re-infused into the arterial compartment. A modern ECLS circuit consists of several components including venous and arterial cannulas, tubing, a membrane oxygenator with gas blender, a continuous-flow centrifugal pump and a heat exchanger to compensate for extracorporeal heat loss (Fig. 1). VA-ECLS was originally derived from cardio-pulmonary bypass (CPB) but differs in several aspects; firstly, CPB uses an open reservoir, whereas VA-ECLS is based on a closed circuit. Secondly, an ECLS circuit actively drains venous blood with negative pressures on the venous side of the pump in contrast to a conventional CPB circuit (using roller pumps) where venous blood is passively drained into a reservoir. Newer CPB circuits, utilising centrifugal pumps, also create a negative pressure on the venous side.

**Fig. 2:**
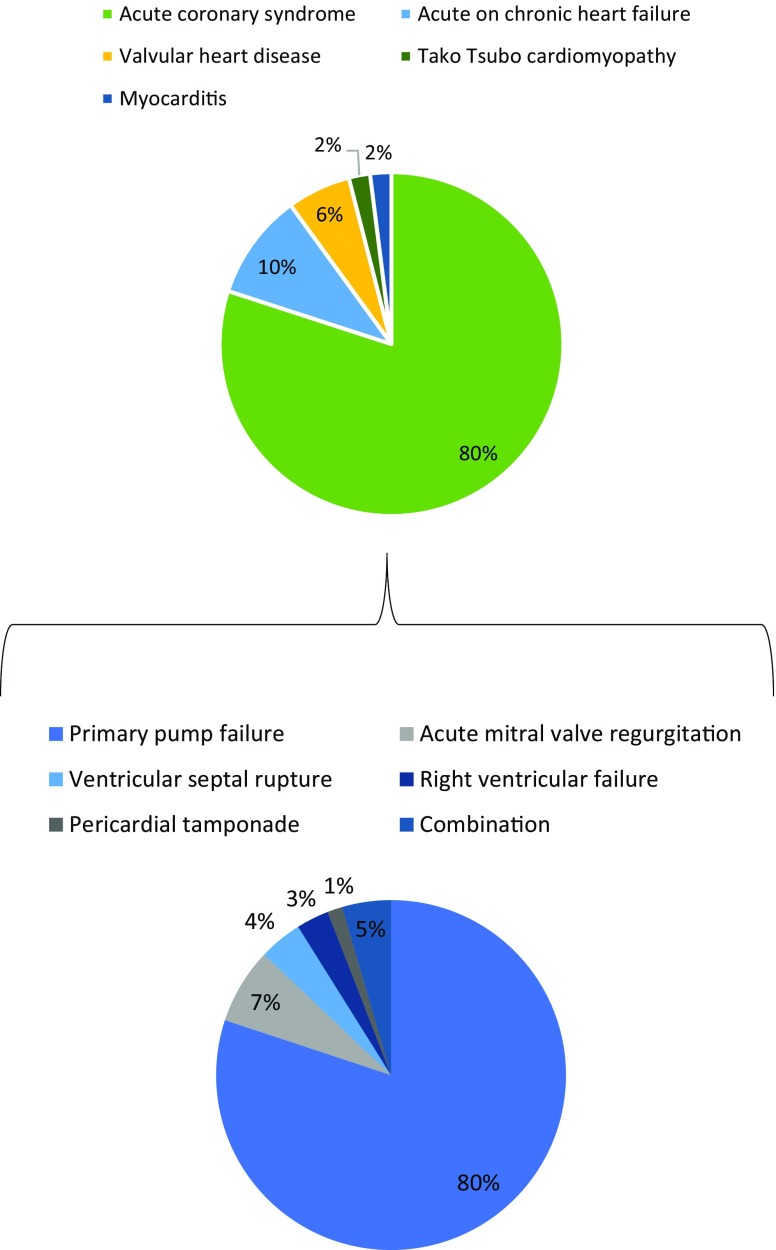
Causes of acute cardiogenic shock in the emergency room

In cardiogenic shock, arterial and venous femoral percutaneous cannulation via the Seldinger technique allows for rapid initiation of VA-ECLS without need for a central approach via sternotomy. Furthermore, it can be performed virtually everywhere inside and outside the hospital environment [11]. For optimal vascular access, echographic assessment can be used, while echo*cardio*graphic and fluoroscopic guidance allows optimal cannula positioning, especially for central venous drainage [12].

Consistent with Poiseuille’s law, narrower, longer cannulas result in larger resistance, which may in turn reduce extracorporeal blood flow or necessitate pressures outside the clinically acceptable range for a given pump speed. Drainage of central venous blood can best be achieved through relatively large 21–25 French (Fr) multistage cannulas. Arterial cannulas are narrower (15–19 Fr) and shorter and allow injection of oxygenated blood retrogradely into the descending aorta. Centrifugal pumps, being used in modern VA-ECLS circuits, are designed to provide a flow of 60–120 ml/kg/min (range of 2–10 l/min). Maximum achievable extracorporeal blood flow depends on many other ECLS circuit and patient characteristics, such as caval vein diameters, volume status, thoraco-abdominal pressures, flow capacity of the oxygenator and maximally tolerable negative and positive pressures (±300 mm Hg). Membrane oxygenators are made up of selectively permeable membranes, facilitating blood and gas flow to allow gas exchange.

## Indications, contra-indications and prognosis

### Indications

The main goal of VA-ECLS in refractory cardiogenic shock is to provide immediate circulatory and respiratory stabilisation, while accounting for sufficient cardiac unloading. Although no randomised controlled trials exist on the effectivity of VA-ECLS, observational studies have shown survival benefits in comparison to conventional therapy and Class IIb and Class IIa recommendations have been specified by European [7] and American [13] guidelines, respectively [10]. To maximise the potential of cardiac recovery and prevent impending multi-organ failure, early initiation of VA-ECLS has been proposed [14]. Observational evidence further suggests that in Interagency Registry for Mechanically Assisted Circulatory Support (INTERMACS) level 1 patients (so called ‘crash-and-burn’ patients), temporary clinical optimisation with VA-ECLS prior to left ventricular assist device (LVAD) support improves outcome as compared to direct implantation of a permanent LVAD [15].

Indications for VA-ECLS can be subdivided according to cause and setting (Tab. 1; Fig. 2). In the emergency room, up to 80% of cardiogenic shock states is caused by acute coronary syndromes with or without ST-segment elevations [16]. Within this subgroup of acute coronary syndromes, cardiogenic shock can be attributed to primary left ventricular (LV) failure in almost 80% of patients. In the other 20%, cardiogenic shock is caused by acute mitral regurgitation (6.9%), septal rupture (3.9%), right ventricular failure (2.8%), and pericardial tamponade (1.4%) secondary to the myocardial infarction [17].

**Table 1:** Indications and contra-indications for VA-ECLS

Indications	Contra-indications
Refractory cardiogenic shock due to:	Absolute
– Acute coronary syndrome (with or without mechanical complications) – Acute valvular heart disease – Acute deterioration of non-ischaemic cardiomyopathy – Acute myocarditis – Tako Tsubo cardiomyopathy – Intractable arrhythmias	– Recent intracranial haemorrhage or infarction – Uncontrolled coagulopathy – Multi-trauma with high risk of bleeding – Irreversible cardiac disease with no prospect for permanent ventricular assist device implantation or heart transplantation – Aortic dissection and severe aortic regurgitation
Refractory cardiac arrest	
Post-cardiotomy cardiogenic shock	Relative
	– Age >65 years

*VA-ECLS* veno-arterial extracorporeal life support

Other common causes of cardiogenic shock suitable for VA-ECLS may relate to acute deterioration of chronic heart failure (10%), valvular disease (6%), stress-induced cardiomyopathy (2%), myocarditis (2%), and other causes such as refractory ventricular tachyarrhythmias (<1%) [18].

Cardiac arrest refractory to conventional cardio-pulmonary resuscitation (CPR) forms another potential indication for VA-ECLS, i. e., extracorporeal-CPR (E-CPR) for in-hospital and out-of-hospital cardiac arrest. Observational studies have suggested a favourable outcome in otherwise refractory in-hospital cardiac arrest [19] and out-of-hospital cardiac arrest [20]. The current European Resuscitation Council (ERC) guidelines also advocate considering E‑CPR under favourable clinical circumstances. At present, E‑CPR remains largely an experimental therapy and further scientific evidence is imperative to clearly identify the subgroup of patients that may truly benefit from such a demanding and costly modality.

VA-ECLS is classically used in post-cardiotomy cardiogenic shock, which occurs in 0.2–6% of cardiac operations [21]. In addition to its use in strictly cardiological and cardio-surgical indications, VA-ECLS has been successfully applied in patients with acute pulmonary embolism [22]. However. evidence remains confined to case reports and limited series. Furthermore. patients with septic shock [23], anaphylaxis [24], severe intoxications [25], or trauma/multi-trauma with circulatory, cardiac and/or respiratory failure have been successfully supported with VA-ECLS [26]. In these patient subgroups with severe combined circulatory and respiratory insufficiency the attribute of gas exchange in VA-ECLS can be of paramount importance.

### Contra-indications

Before initiation of VA-ECLS, several absolute and more relative contra-indications should be considered (Tab. 1). Firstly, VA-ECLS should serve as a bridge to cardiac recovery or long-term mechanical support. In patients without such prospects, VA-ECLS should not be used. A significantly reduced life expectancy due to non-cardiac morbidities (e. g. metastasised malignancies or severe pulmonary disease) should also serve as a reason to refrain from VA-ECLS. Furthermore, uncontrollable coagulation disorders or intracranial bleeding should be interpreted as absolute contra-indications. Other arguments relate to anatomical or disease-related constraints to the insertion of VA-ECLS cannulas such as aortic dissection (prior to surgical correction), extensive multi-trauma, or peripheral artery disease. In patients with severe aortic regurgitation VA-ECLS should not be used because of an uncontrollable increase in regurgitant volume. Older age is a relative contra-indication although limits are ill-defined.

### Prognostication

According to the Extracorporeal Life Support Organization (ELSO) registry, overall in-hospital survival of patients treated with VA-ECLS approximates 40% [27]. Patients with myocarditis have a relatively better prognosis (in-hospital survival of 62%), whereas patients with congenital defects demarcate the poorer end of the spectrum showing average survival rates of 37%. Patients with non-ischaemic heart disease have a better prognosis than those with ischaemic cardiomyopathy. In cardiac resuscitation, VA-ECLS has roughly been associated with a 30% hospital survival only.

For assessment of patient-specific prognosis, several scores have been developed. The Survival After Veno-arterial Extracorporeal membrane oxygenation (SAVE) score (Tab. 2), available online at www.save-score.com, was based on 3,846 patients from the ELSO registry and revealed satisfactory performance at external validation [28]. The theoretical risk distribution varies between 10 and 100%, depending on the combination of clinical characteristics as outlined in Tab. 2 and detailed in the original publication [28]. The ENCOURAGE score, which was developed based on 138 patients, claimed to have higher discriminatory abilities than the SAVE score [29]. Finally, the INTERMACS classification was designed for patients receiving long-term LVADs [30]. With patients qualifying for VA-ECLS already in a poor clinical condition, reflected by an INTERMACS level 1 or 2, this score does not seem to add additional discriminative power.

**Table 2:** SAVE score variables

Variables	Input
*Diagnosis*	
– Myocarditis	Yes/no
– Refractory VT/VF	Yes/no
– Post heart/lung transplantation	Yes/no
– Congenital heart disease	Yes/no
– Other	Yes/no
*General*	
– Age	Categories; 18–38, 39–51, 53–62 years
– Weight	Categories: <65, 65–89, ≥90 kg
*Cardiac*	
– Pulse pressure ≤20 mm Hg^a^	Yes/no
– Diastolic blood pressure ≥40 mm Hg^a^	Yes/no
– Cardiac arrest	Yes/no
*Respiratory*	
– Peak inspiratory pressure 20 cmH2O	
– Intubation duration	Categories: ≤10, 11–29, ≥30 hours
*Renal*	
– Acute renal failure^b^	Yes/no
– Chronic renal failure^c^	Yes/no
– HCO3 pre-ECLS <15 mmol/l^d^	Yes/no
*Other organ failures*	
– Central nervous system dysfunction^e^	Yes/no
– Liver failure^f^	Yes/no

*ECLS* extracorporeal life support,* eGFR* estimated glomerular filtration rate,* VT* ventricular tachycardia, *VF* ventricular fibrillation

^a^Worst value within 6 h prior to cannulation

^b^Creatinine levels >133 µmol/l (5 mg/dl)

^c^Kidney damage or eGFR <60 ml/min/1.73 m^2^ for ≥3 months

^d^Worst value before cannulation

^e^Neurotrauma, stroke, encephalopathy, cerebral embolism, seizure and epileptic syndromes

^f^Bilirubin ≥33 mcmol/l or elevation of serum aminotransferases (ALT or AST) >70 UI/l at ECLS cannulation

All values prior to cannulation

## Complications

A broad spectrum of complications can be encountered during VA-ECLS. These complications are described below.

**Fig. 3:**
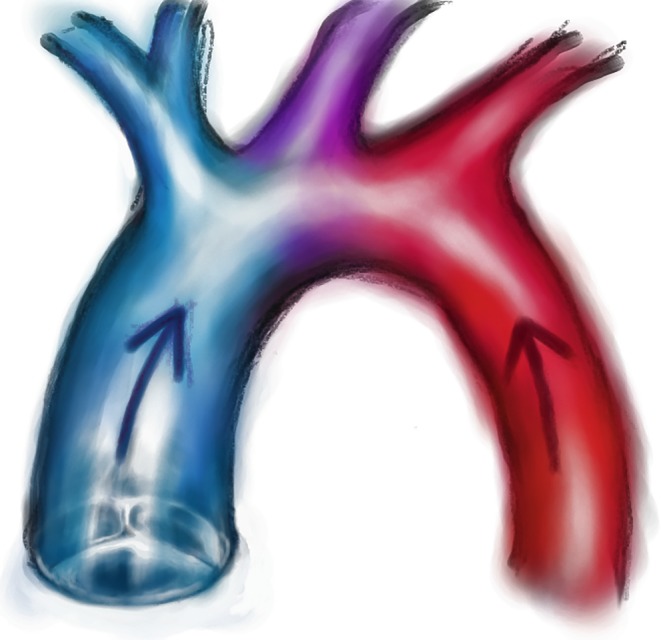
Harlequin’s syndrome. Harlequin’s syndrome can arise when relatively de-oxygenated blood, ejected by the left ventricle in case of poor pulmonary oxygenation, perfuses the aortic arch

- *Vascular complications:*
  These include vascular dissection or perforation upon cannulation, potentially resulting in ischaemia and compartment syndrome of the lower extremities. Rarely, perforation of the right atrium may occur which underscores the importance of periprocedural trans-oesophageal echocardiographic guidance [31]. Next, limb ischaemia is often seen as a consequence of cannula-related impediment of arterial blood flow distal to the cannulation site [32]. Compression of the vein may also compromise venous blood flow. Therefore, it is imperative to closely monitor leg ischaemia and, if suspected, adequate measures should be taken immediately. These measures include distal cannulation using a reinforced sheath connected to the arterial limb of the extracorporeal circuit or a ‘chimney construction’ using a T-shaped Dacron tube with bi-directional arterial exit. These preventive steps should be considered in the use of relatively large-sized cannulas or in unilateral veno-arterial cannulation [32]. Ischaemic limb complications occur in less than 5% of cases and can often be dealt with in an adequate way but should be recognised in time and not be underestimated.
- *Haemodynamic complications:*
  Being counterintuitive at first, VA-ECLS increases LV afterload due to continuous, retrograde infusion of arterialised blood into the descending aorta. This, in turn, creates elevated LV end-diastolic pressures and consequently may cause pulmonary oedema. When LV overload is not timely recognised, cardiac function and ventricular mechanics may further deteriorate. Therefore, close monitoring of an adequate pulsatility of peripheral arterial pressure tracings (as a measure of LV ejection) is imperative. Next, close echocardiographic follow-up of cardiac geometry and function is imperative to prevent irreversible cavity dilatation and progressive loss of LV contractility. The clinical significance of pulmonary oedema in VA-ECLS is underscored by its incidence exceeding 30% of supported patients and its potential to deteriorate into acute lung injury (ALI), which in turn limits long-term outcome even after initially successful bridging to chronically implanted ventricular assist devices [33].
- *Thromboembolic complications:*
  The ELSO registry reported a high incidence of 0.5 thrombotic events per case, but this is likely underestimated [34]. Formation of thrombo-emboli is in part caused by contact of blood with the artificial surfaces of the VA-ECLS circuit. Thrombi can form virtually everywhere in the circuit, grow, migrate and may even manifest after decannulation [35]. The absence of aortic valve opening may cause LV cavity and left atrial or aortic root thrombosis [36]. In order to minimise the risk of thrombus formation, anticoagulation, classically unfractionated heparin, is generally advocated during VA-ECLS and biocompatible heparin-coated surfaces are widely used.
- *Bleeding:*
  Bleeding may arise at various locations like the cannulation site, lungs, gastro-intestinal tract, but can also occur in the pericardium (causing tamponade), or intra-cranially. The incidence rate is described as being comparable to thromboembolic events (0.5 events per patient treatment) but can become significantly higher in certain settings such as post-cardiotomy syndrome or after lung transplantation. The pathophysiology of coagulation disorders in VA-ECLS is extremely complex and includes direct and indirect effects of anticoagulants, haemolysis, thrombocytopenia, loss of coagulation factors and the continuous-flow itself. Controversies exist about the true clinical significance of acquired von Willebrand disease and the incidence of heparin-induced thrombocytopenia, which seems to be rather low [37–39].
- *Cerebrovascular events:*
  A cerebrovascular accident is one of the most devastating complications and may occur as a consequence of thrombotic or air emboli or due to bleeding [1]. The latter could also be superimposed on an ischaemic event. Cerebral ischaemia can also arise due to perfusion with predominantly desaturated blood, a phenomenon referred to as ‘Harlequin’s syndrome’. As illustrated in Fig. 3, well-oxygenated blood from the membrane oxygenator, is infused retrogradely into the descending aorta and usually mixes with blood flow from the heart in the aortic arch. When significant pulmonary dysfunction prevents adequate oxygenation of blood in the pulmonary circulation; the aortic arch, upper right extremity and cerebrum may receive deoxygenated blood from the left ventricle with potentially deleterious consequences. Therefore, mechanical ventilation, if needed, should always be titrated to an adequate positive end-expiratory pressure (PEEP) (and oxygen level) to assure optimal pulmonary capillary oxygenation. In order to timely detect this phenomenon, continuous pulse oximetry and repeated arterial blood gas analyses from the right arm should be monitored closely as they best reflect cerebral oxygenation via the proximal aortic arch. Finally, cerebral hypoperfusion may result from vascular spasm, promoted by, for example, hypocapnia due to inadequate gas flow management. Currently, the complex brain-ECLS interactions are far from understood and deserve specific attention in clinical practice [40].
- *Systemic inflammation:*
  Systemic inflammation as a result of blood contact with artificial surfaces, infectious complications and multiorgan failure are well-documented, feared complications [27]. Infection (e. g. cannulation site infections, bacteraemia, pneumonia) is one of the most common complications in VA-ECLS occurring in up to 13% of adult patients [27]. In this context physicians should realise that ongoing culture-negative systemic inflammation may indicate membrane oxygenator colonisation which should prompt its immediate replacement [41]. The physician should also take into consideration that significantly altered pharmacokinetics during VA-ECLS may result in sub-therapeutic levels of antibiotics [42].
- *Renal complications:*
  Acute kidney injury is common in patients on VA-ECLS, but the reported incidence estimates vary greatly (roughly 10–85%) throughout studies [27, 43], in part because of inconsistencies in definitions of acute kidney injury. A considerable subset of patients even requires continuous renal replacement therapy (CRRT), which is reported to have a significant adverse impact on outcome [43, 44].

## Management

VA-ECLS is a technically sophisticated and expensive treatment. Adequate management requires expert knowledge of the extracorporeal circuit, cardiac and vascular mechanics, as well as mechanical ventilation, coagulation, and infectious diseases. This implies a 24-hour multidisciplinary coverage of trained nurses, residents, intensivists, cardiac-surgeons, cardiologists, anaesthesiologists and perfusionists. Systematic monitoring should include regular (hourly) check-ups to screen for potential complications such as systemic emboli, bleeding, infection, Harlequin’s syndrome and vascular problems and, last but not least, adequate systemic perfusion and LV unloading.

### Left ventricular unloading

The holy grail of supporting patients in cardiogenic shock with VA-ECLS is to find an optimal balance between LV unloading, sufficient residual flow through the pulmonary circulation and left ventricle, while simultaneously providing adequate systemic perfusion. Similar to regular heart failure care, pre- and afterload should be kept as low as possible. In order to lower preload, circulating volume should be condensed as much as possible while preventing suction events because they reduce flow and may cause cavitation. A low afterload is imperative, but must be balanced against the maintenance of an adequate systemic perfusion pressure to ensure optimal organ perfusion. Lowering systemic blood pressure can be achieved by administration of afterload-lowering medication, e. g., sodium nitroprusside and nitroglycerin. On the other hand, during vasoplegia, noradrenaline may be necessary.

A general VA-ECLS management strategy should aim at tailoring extracorporeal blood flow to the lowest degree possible, just exceeding a critical flow preventing circuit thrombosis (typically >2 l/min). This so called *partial support strategy* allows the use of small-sized cannulas (15–17 Fr). The sum of VA-ECLS flow and native cardiac output (where the aortic valve opens and a dicrotic notch is visible) should still meet physiological and/or pathophysiological requirements. When these targets cannot be met, inotropic drugs such as dobutamine and milrinone can be used. When all of these measures provide too little LV unloading, interventional strategies should be considered. Although routine use of the IABP in conjunction with VA-ECLS is not advised [44], a mild, but sometimes essential, reduction of pulmonary capillary wedge pressures may be achieved [45]. Alternative approaches providing more potent LV unloading are:

1. simultaneous use of VA-ECLS and a transaortic, axial device (Impella®);
2. the creation of an interatrial septostomy to enable shunting of blood from the left to the right heart;
3. direct surgical venting with a vent from the pulmonary artery or from the left ventricle using a temporary cannula connected to the venous side of the ECLS circuit [46, 47].

In this latter system, a so-called hybrid 1 ½ VAD ECLS circuit is created.

### Weaning

Weaning from VA-ECLS is one of the greatest challenges in daily management and should already be considered early in the clinical course of support. Signs of improving cardiac function during VA-ECLS include increasing blood pressures (typically mean arterial pressure >65 mm Hg), increasing pulsatility of the arterial pressure waveform, and reduced need for inotropics and vasopressors. Several echocardiographic parameters have been proposed to assess recovery of LV function on reducing the VA-ECLS flow, including ejection fraction >30%, and aortic Velocity Time Integral (VTI) >10 cm [48]. Biomarkers to reflect cardiac loading conditions [49] and strain imaging have so far not conveyed advantages in VA-ECLS management [50].

Removal of VA-ECLS must be considered as soon as the patient has been clinically stable for 24 h, the inotropic demand is low and significant fluid overload is absent. Prior to extraction of the VA-ECLS circuit, a weaning trial is performed consisting of reducing pump flow to e. g. 50% or 1 l/min under adequate anticoagulation. When haemodynamics are maintained in a stable and adequate condition extraction can be considered after administration of protamine [51]. In most patients, successful weaning can be performed between day 2 and day 5 after treatment initiation, although this clearly will depend on the nature and severity of the underlying disease and its individual clinical course.

### Strategies when cardiac function does not recover

When recovery of ventricular function fails to occur or is deemed unlikely, a decision to withdraw treatment, or to initiate a long-term support strategy is required. Long-term treatment options are practically confined to implantation of a continuous-flow LVAD, while urgent cardiac transplantation remains preserved for only a few selected cases. Ideally, a patient would be awake to take part in this decision making, which is best possible after detubation while on VA-ECLS, but requires optimal tailoring of extracorporeal blood flow and gas exchange [52, 53]. Importantly, LVAD implantation requires a sufficiently preserved right ventricular function, since long-term mechanical support in right ventricular or biventricular failure carries an unfavourable prognosis.

## Conclusions

VA-ECLS is an increasingly used cardiac and circulatory support modality, which can provide immediate stabilisation in patients with otherwise refractory cardiogenic shock. In the absence of randomised trials, observational studies have suggested a reduction in mortality as compared to conventional treatment. VA-ECLS should be taken into consideration in well-selected cases. Management of VA-ECLS is complex and requires constant support of trained personnel. For this reason, the application of this support technique should be confined to centres with sufficient experience, ongoing exposure and a close and well-organised multidisciplinary team.
